# Mass spectrometry data from a quantitative analysis of protein expression in gills of immuno-challenged blue mussels (*Mytilus edulis*)

**DOI:** 10.1016/j.dib.2016.05.073

**Published:** 2016-06-03

**Authors:** K. Hörnaeus, J. Guillemant, J. Mi, B. Hernroth, J. Bergquist, S. Bergström Lind

**Affiliations:** a Department of Chemistry – BMC, Analytical Chemistry and SciLifeLab, Uppsala University, Box 599, SE – 75124 Uppsala, Sweden; b The Royal Swedish Academy of Sciences, Sven Lovén Center for Marine Science, Kristineberg 566, SE – 451 78 Fiskebäckskil, Sweden; c Department of Natural Science, Kristianstad University, SE – 291 88 Kristianstad, Sweden

**Keywords:** Mass spectrometry data, Blue mussels, Mytilus edulis, Gills, Quantitative proteomics

## Abstract

Here, we provide the dataset associated with our research article on the potential effects of ocean acidification on antimicrobial peptide (AMP) activity in the gills of *Mytilus edulis*, “Impact of ocean acidification on antimicrobial activity in gills of the blue mussel (*Mytilus edulis*)” [1]. Blue mussels were stimulated with lipopolysaccharides and samples were collected at different time points post injection. Protein extracts were prepared from the gills, digested using trypsin and a full in-depth proteome investigation was performed using liquid chromatography coupled to tandem mass spectrometry (LC–MS/MS). Protein identification and quantification was performed using the MaxQuant 1.5.1.2 software, “MaxQuant enables high peptide identification rates, individualized p.p.b.-range mass accuracies and proteome-wide protein quantification” [2].

**Specifications Table**

**Table:** 

Subject area	*Biology*
More specific subject area	*Protein expression in Mytilus edulis*
Type of data	*Table,.xlsx file*
How data was acquired	*Data dependent LC-MS/MS acquired on a Q Exactive instrument (Thermo Fisher Scientific) coupled to a ultra nanoflow high-performance liquid chromatography (HPLC) system (EASY-nLC™ 1000, Thermo Fisher Scientific)*
Data format	*Processed, analyzed*
Experimental factors	a) *Lipopolysaccharide stimulation of blue mussels and protein extraction from gills*
	a) *LC-MS/MS analysis for protein identification and quantification*
Experimental features	*Blue mussels were stimulated with lipopolysaccharides and samples were collected at different time points post injection. Protein extracts were prepared from the gills, digested using trypsin and a full in-depth proteome investigation was performed using LC-Orbitrap MS/MS technique. The spectra (.RAW) were acquired using Xcalibur software 3.0.63 and further database searches were performed using MaxQuant* 1.5.1.2. *The search results were stored as.xls-files.*
Data source location	*Uppsala, Sweden*
Data accessibility	*Data are within this article* (Supplementary Table 1)

**Value of the data**

• The data further validate the protein expression changes presented in Hernroth et al. [1].
• The data can be used to validate protein identification in *Mytilus edulis* from other studies.
• The in depth proteomic data enables comparison with RNA expression data.

## 1 Data

This dataset comprise the output file (Supplementary Table 1, available online) from the database search of LC–MS/MS raw files obtained from bottom-up MS analysis of gills from *Mytilus edulis* immune-challenged by lipopolysaccharide injection. Samples were collected at five time points post injection of lipopolysaccharide (Table 1). One control group of mussels injected with only *Mytilus* physiological saline (PS)-buffer was included. Each group included five individual mussels.

## 2 Experimental design, materials and methods

### 2.1 Experimental set up

Thirty mussels were kept in the running seawater system of SLC-Kristineberg (~32 PSU, 14 °C) and divided into six 15 L basins with five individuals in each. Bacterial contamination was avoided by pre-challenging with lipopolysaccharide (LPS; #L7261, Sigma Aldrich) dissolved in PS-buffer [3]. One control group of mussels was injected with only PS-buffer and the other five groups were injected with 0.2 µg LPS g^−1^ mussel (wet weight), into the adductor muscle. The gills of mussels from the control group were dissected at time 0 followed by dissection of one group at a time after 0.5, 1.5, 3, 5 and 8 h post injection. The dissected gill tissues were immediately put on dry ice before being stored at −80 °C until further prepared.

### 2.2 Sample preparation

The samples were homogenized in 9 M urea, 20 mM HEPES using a micro pestle. Proteins were extracted by sonication using a probe with a 3 mm tip (10×1 s, amplitude 30%) followed by centrifugation at 16,000*g* for 20 min at 4 °C. The total protein concentration in the samples was analyzed using Coomassie assay, with bovine serum albumin (BSA) as standard [4]. Aliquots corresponding to 20 μg protein were reduced with dithiothreitol (DTT) and alkylated with iodoacetamide (IAA). After four times dilution with 50 mM ammonium bicarbonate, trypsin was added in a trypsin:protein ratio of 1:20 and digestion was performed overnight. Thereafter the samples were purified by Pierce C18 Spin Columns (Thermo Scientific), dried and resolved in 60 µL 0.1% formic acid.

### 2.3 LC–MS/MS

The samples were analyzed using a QExactive Plus Orbitrap mass spectrometer (Thermo Fisher Scientific, Bremen, Germany) equipped with a nano electrospray ion source. The peptides were separated by reversed phase liquid chromatography using an EASY-nLC 1000 system (Thermo Fisher Scientific). A set-up of pre-column and analytical column was used. The pre-column was a 2 cm EASY-column (1D 100 µm, 5 µm C18) (Thermo Fisher Scientific) while the analytical column was a 10 cm EASY-column (ID 75 µm, 3 µm, C18; Thermo Fisher Scientific). Peptides were eluted with a 90 min linear gradient from 4% to 100% acetonitrile at 250 nL min^−1^. The mass spectrometer was operated in positive ion mode acquiring a survey mass spectrum with resolving power 70,000 (full width half maximum), m/z = 400-1750 using an automatic gain control (AGC) target of 3×10^6^. The 10 most intense ions were selected for higher-energy collisional dissociation (HCD) fragmentation (25% normalized collision energy) and MS/MS spectra were generated with an AGC target of 5×10^5^ at a resolution of 17,500. The mass spectrometer worked in data-dependent mode.

### 2.4 Mass spectrometry data handling

The acquired data (.RAW-files) were processed by MaxQuant 1.5.1.2 [2] and database searches were performed using the implemented Andromeda search engine. MS/MS spectra were correlated to a FASTA database containing proteins from *Mytilus* extracted from the NCBI database (release June 2015). A decoy search database, including common contaminants and a reverse database, was used to estimate the identification false discovery rate (FDR). An FDR of 1% was accepted. The search parameters included: maximum 10 ppm and 0.6 Da error tolerances for the survey scan and MS/MS analysis, respectively; enzyme specificity was trypsin; maximum one missed cleavage site allowed; cysteine carbamidomethylation was set as static modification and oxidation (M) was set as variable modification. For protein identification, only peptides with a minimum of seven amino acids and at least one unique peptide were accepted.

## Figures and Tables

**Table 1.** Samples included for each time point post injection.

**Sample number**	**Time post injection (h)**
1–5	Ctrl
6–10	0.5
11–15	1.5
16–20	3
21–25	5
26–30	8
